# Supplementary material for: Relationship between Serum Kallistatin and Afamin and Anthropometric Factors Associated with Obesity and of Being Overweight in Patients after Myocardial Infarction and without Myocardial Infarction
Source: J Clin Med. 2021 Dec 10;10(24):5792. doi: 10.3390/jcm10245792 (PMC8708718; doi:10.3390/jcm10245792)
Supplement: Supplementary file 1 [file jcm-10-05792-s001.zip › jcm-1490014-supplementary.pdf]

Supplementary Materials

Relationship Between Serum Kallistatin and Afamin and Anthropometric Factors Associated with Obesity and Overweight in Patients After Myocardial Infarction and Without Myocardial Infarction

Grzegorz Józef Nowicki, Barbara Ślusarska, Maciej Polak, Katarzyna Naylor and Tomasz Kocki

Table S1. Laboratory test results in the studied groups.

**Variables**	**Studygroup (*n* = 80)**	**Control group (*n* = 80)**	***p***
	Laboratory tests:		
Total cholesterol [mg/dL]	147.8 ± 37	221.9 ± 44.2	<0.001
HDL-C [mg/dL]	46.2 ± 11.5	64.05 ± 19.14	<0.001
non-HDL [mg/dL]	101.5 ± 34.31	157.9 ± 47.22	<0.001
LDL-C [mg/dL]	70.2 ± 25.86	134.81 ± 42.37	<0.001
Triglyceride [mg/dL]	134.5 (103.25 - 174.35)	102.91 (73.41 - 141.15)	<0.001
Glucouse [mg/dL]	102.51 (97.01 - 112.01)	102 (97.51 - 109.51)	0.08
Creatinine [mg/dL]	0.85 ± 0.15	0.86 ± 0.18	0.97
eGFR [mL/min/1.73 m2]	93.52 ± 12.56	95.4 ± 13.33	0.35

Data are presented as means ± standard deviation (SD) or median (interquartile range). *p* value in this table was analyzed between 2 groups; Abbreviations: HDL-C, high-density lipoprotein; LDL-C, low-density lipoprotein; eGFR, estimate glomerular filtration.
